# Microbial cement grout anchoring steel reinforcement pull-out test

**DOI:** 10.1038/s41598-024-52196-z

**Published:** 2024-01-18

**Authors:** Qiang Jia, Tianjian Zhang

**Affiliations:** 1 School of Civil Engineering, Shandong Jianzhu University, Jinan, 250100 Shandong China; 2 Key Laboratory of Building Structural Retrofitting and Underground Space Engineering (Shandong Jianzhu University), Ministry of Education, Jinan, 250100 Shandong China

**Keywords:** Civil engineering, Biological techniques, Engineering

## Abstract

Microbial cement, known for its superior fluidity, stable crystal formation, and strong bond with concrete, is an effective solution in fixing defects such as voids that appear due to insufficient grouting in the joints of precast concrete components. To evaluate the mechanical properties of rebar anchored with microbial cement grout, three pull-out tests were designed, taking into account parameters such as calcium source concentration, the filler in borehole voids, and the shape of the rebar. The results indicate that specimens with a higher concentration of calcium source require fewer grouting cycles, but the pull-out bearing capacity of the anchored rebar is lower. However, the introduction of quartz sand as a filler in the borehole voids results in a significant increase in the pull-out bearing capacity of the rebar compared to specimens without filler. Among these, the specimens with a medium particle size of 0.5 ~ 1 mm (1/4*δ* ~ 1/2*δ*, the gap of *δ* = 2 mm between the steel bars and hole wall) have the highest pull-out bearing capacity. In comparing rebar shapes, ribbed rebar slightly outperforms smooth round rebar in terms of pull-out bearing capacity. Based on the experimental results analysis, the anchoring mechanism of microbial cement grouting on reinforcement has been elucidated.

## Introduction

The prefabricated component model of assembled buildings boasts advantages such as energy conservation, environmental protection, rapid production cycles, and labor-saving. Currently, the vertical components of concrete structures in assembled buildings are typically connected using grout sleeves or mortar anchors, with the connection usually achieved through grout injection. However, in practical engineering, there are often instances where the grout is not fully filled, leading to internal voids in the connections. This results in insufficient anchoring length for the grout on the rebar or reduced bond strength, subsequently affecting the mechanical and durability properties of the connection^1^. It proves quite challenging to re-grout these voided joints using traditional cement-based grout. Furthermore, the commonly used grout, which is a regular cement-based material, also has drawbacks such as high energy consumption and environmental pollution. Therefore, it is imperative to develop a novel, sustainable cementitious material to address these challenges presented by traditional grouting materials in practical engineering. The emergence of microbial cement offers a fresh approach to solving these issues. This material has high fluidity before it mineralizes and can effectively seal internal voids in the grout under pressure. Additionally, microbial cement is produced through urease formed by microbial metabolism, a natural and eco-friendly process. The composition of microbial cement is calcite, which has a stable crystal form and pairs well with concrete, thus significantly improving the mechanical properties of the connected components after repair. Therefore, the application of microbial mineralization deposition technology in the connection of concrete components is highly significant. Many experts and scholars domestically and internationally have conducted extensive research on the mechanical properties of microbial cement.

Over the years, researchers globally have extensively explored the mechanical properties of microbial cement. Kantzas et al., as early as 1992, began investigating the technology of microbial induced calcium carbonate deposition during their research on new cementitious materials. They successfully applied this technology to consolidate sand^2^. In a pioneering study, Gollapudi mixed anaerobic bacteria with sand to obtain sand columns possessing a certain strength. This study confirmed that microorganisms can induce the formation of calcium carbonate precipitation^3^. Lee et al. incorporated microbial cement into residual soil, resulting in precipitation that caused the consolidation of the residual soil. Through the analysis of stress–strain and compression characteristic curves of the specimen post microbial treatment, it was deciphered that the residual soil, post microbial cement consolidation, exhibits typical brittle failure characteristics^4,5^. Furthermore, Lee et al. enhanced the grouting process of residual soil by modulating the concentration of different bacterial liquids, the grouting liquid concentration, and altering the grouting pressure, leading to an increase in the shear strength of the solidified soil by up to 100%^6^. Kim et al., through their study on the consolidation effect of microbial grouting in different soil types, deduced that sand and silt soils are most favorable for the relative compactness of calcium carbonate precipitation. They also discovered a significant correlation between the amount of calcium carbonate precipitation and the gaps in the soil^7^. Zhao et al. conducted a comprehensive study on the influence of bacterial concentration, cement concentration, and reaction time on microbial-induced calcium carbonate precipitation and consolidation within sand bodies. The experiments revealed that factors such as the concentration of bacterial solution, cementitious solution, and reaction time significantly enhance the strength of the sand column post microbial sand solidification, while the curing conditions exert minimal impact on its performance^8^. Meanwhile, Hill et al. employed microorganisms and nutrients to fill the gaps in fractured rocks, discovering that the fractures in the broken rock can effectively accommodate the mineralized sediment induced by microorganisms. This process notably enhances the impermeability of the rock mass^9^. In a different context, Levrel et al. adopted microbial-induced calcium carbonate precipitation technology for building restoration and preservation of historical heritage, gradually extending its application to the repair of concrete materials^10^. Wiktor et al. utilized calcium lactate as a calcium source for repairing cracks. They employed porous lightweight aggregate as a carrier for the calcium source and bacteria to mend cracks ranging from 50 μm to 1 mm in width. The results were promising, with the maximum repair width reaching 470 μm^11^.

Whiffin conducted a bio-calcium carbonate cement experiment, employing a plastic syringe as the mold for cementation. The injection of microbial cementing liquid into the loose sand resulted in a microbial sand body with a shear strength of 1.8 Mpa^12^. In a separate study, Zhang et al. investigated the interfacial shear behavior of MICP-treated calcareous sand and steel under varying levels of cementation and normal stress. They observed an increase in shear thickness band with increasing normal stress, which eventually stabilized^13^. Qian Chunxiang, on the other hand, mixed bacteria solution, substrate solution, and calcium source solution into sandy soil. This process gradually cemented the loose sand particles into a solid mass, which demonstrated a strength of up to 2 MPa in an unconfined compressive test^14^. Jia Qiang proposed a method of using microbial grouting to anchor steel bars in concrete structures. However, mechanical performance indicators for the anchored steel bars were not provided^15^.

The findings from the above research indicate that microbial cement exhibits excellent performance in crack filling. By introducing microbes into the medium and using it as a filler in concrete structures, cracks can be effectively sealed. Additionally, the method of using microbial cement to anchor steel bars has been found feasible. However, this technique is currently at the experimental stage, and further research is necessary to refine the microbial grout anchoring technique for steel bars. In this study, we conducted pull-out tests for steel bars anchored with microbial cement grouting, revealing the internal force distribution of steel bars anchored with microbial cement grouting. Through data analysis, we elucidated the anchoring mechanism of the steel bars by microbial cement grouting.

## Materials and methods

### Test parameters

To investigate the influence of different factors on the pull-out mechanical properties of steel bars anchored with microbial cement grouting, this experiment proposes three different test parameters: (1) Calcium source concentrations of 2.0 mol/L, 1.0 mol/L, and 0.5 mol/L were selected for grouting. (2) This parameter involved the presence or absence of fillers in the anchor hole, as well as the particle size of the filler. The filler used in the experiment was quartz sand. Given the gap of *δ* = 2 mm between the steel bars and hole wall used in the experiment, the filler particle size ranges were set at 0 ~ 0.5 mm (< 1/2*δ*), 0.5 ~ 1 mm (1/4*δ* ~ 1/2*δ*) and 1 ~ 2 mm (1/2*δ* ~ *δ*). The particle size parameters of the standard sand are shown in Table 1. (3) The type of steel bar, with options being smooth round steel bars and ribbed steel bars.The experiment was conducted using a single variable design, with three control groups designed for comparison. The specific design parameters are shown in Table 2.

**Table 1:** Particle size parameters of the three media.

Size range/mm	Average particle size/mm	Uniformity coefficient C_u_
0.000 ~ 0.500	0.31	3.35
0.500 ~ 1.000	0.82	1.24
1.000 ~ 2.000	1.43	2.94

**Table 2:** Table of variable parameters for microbial cement grouting anchorage reinforcement test.

group	Variable parameters	parameter value	Invariant parameters
1	Calcium source concentration	2.0 mol/L	Medialess, ribbed steel bar
		1.0 mol/L	
		0.5 mol/L	
2	Medium particle size	< 0.5 mm	The calcium source concentration is 1.0 mol/L, ribbed steel bar
		0.5 ~ 1 mm	
		1 ~ 2 mm	
3	Rebar shape	Plain round steel bar ribbed steel bar	The calcium source concentration is 1.0 mol/L, Medialess

The concrete base material used to anchor the steel bars has a strength grade of C30 and was cast into cubic test pieces measuring 300 mm × 300 mm × 300 mm. The average measured compressive strength (f_cu_) of the cubes was 33.4MPa. The anchor holes, which were drilled through the test pieces, had a diameter of 18mm. Two types of steel bars were used for anchoring: HPB300 smooth round steel bars and HRB335 ribbed steel bars, both with a diameter of 14mm. Tensile tests were conducted on three sets of each type of steel bar, with the average yield strength and tensile strength recorded in Table 3. The anchored steel bars were inserted to a depth of 280mm into the holes. To prevent bacterial flow during grouting, the hole was sealed with a cotton ball that had been stripped of its fats, which also served to filter the bacteria.

**Table 3:** Mechanical performance test results of reinforcement.

Number	Average yield strength/MPa	Average tensile strength/MPa
HPB300	329.4	437.6
HRB335	363.7	512.3

### Configuration of grout

Microbial cement grouting, based on the technology of microbially induced calcium carbonate precipitation, includes components such as bacterial solution, nutrient salt solution, and calcium source solution.

#### Configuration of bacterial liquid

**Figure 1:**
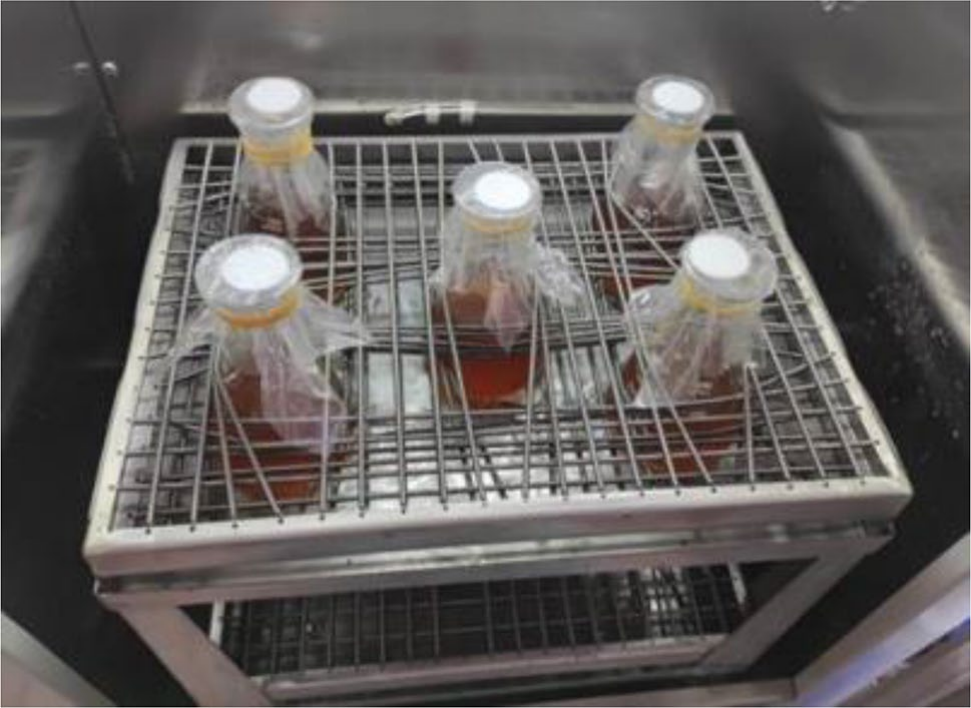
Constant temperature shaker incubator.

The bacterial strain selected for this experiment was Bacillus Pasteurii, which exhibits stable and effective calcium carbonate precipitation. The culture medium consisted of yeast extract as the organic carbon source with a concentration of 20 g/L, ammonium sulfate as the organic nitrogen source at a concentration of 10 g/L, and nickel chloride as a growth factor at a concentration of 10 μmol/L. The steps for the bacterial solution culture included: weighing and dissolving the medium, adjusting the pH of the culture medium, sterilizing the culture medium under high temperature and pressure, inoculating the bacteria, culturing in a constant temperature shaker incubator, and measuring the enzyme activity. The constant temperature shaker incubator is shown in Fig. 1. A total of six grouting cycles were conducted, thus six batches of bacterial solutions were cultured, each with an enzyme activity above 8 mM/min, as shown in Table 4.

**Table 4:** Enzyme activity of bacterial liquid.

Culture batches	1	2	3	4	5	6
Activity (mM/min)	9.55	9.33	9.11	8.88	8.88	8.66

#### Configuration of nutrient salt solution

The nutrient salt used in the experiment was a urea solution. According to the optimized experiments carried out by the project group, the concentration of urea was set at 2.5 mol/L. To enhance the yield of CaCO3 precipitation, the urea solution was added to the bacterial solution one hour before grouting commenced^16^. The volume ratio of the bacterial solution to urea was 1.5:1.

#### Calcium source solution

The calcium source employed in the experiment was a calcium nitrate solution. Its concentration was selected as an experimental parameter, with values of 2.0 mol/L, 1.0 mol/L, and 0.5 mol/L. During the grouting process, the volume ratio of the calcium source solution to the mixture of bacterial solution and urea was 1.5:1.

### Grouting method

A grouting cycle consists of the following steps: First, the bacterial solution is mixed with the urea solution, followed by an interval of two hours before the addition of the calcium source solution. During the injection process, ensure a uniform speed to attain an optimal fusion of the bacterial solution, urea mixture, and calcium source. After each grouting cycle, the resulting mixture is allowed to settle for three days to stabilize the precipitates before embarking on the next grouting cycle. After each cycle, a set of samples is reserved for tensile strength testing.

**Figure 2:**
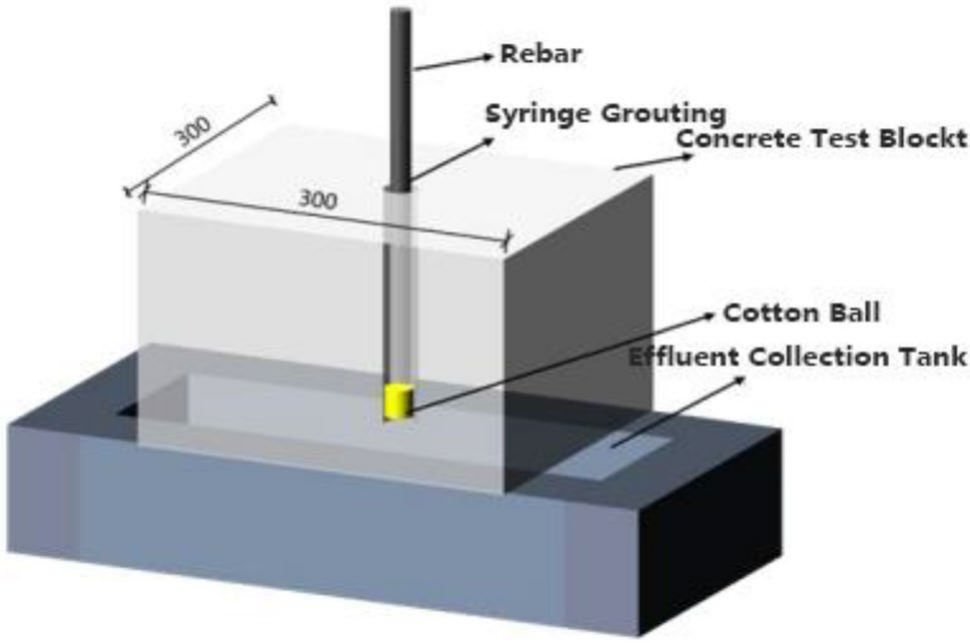
Schematic diagram of the grouting device.

Each grouting cycle involves the injection of a calcium source solution volume nine times the volume of the borehole, approximately 640 mL, and the volume of the bacterial and urea solution six times the borehole volume, about 427 mL. The grout fluid flowing out from the bottom of the hole is considered waste and is not reused. A schematic diagram of the grouting apparatus is shown in Fig. 2.

### Test scheme for resist pull-out bearing capacity of anchored steel bars

**Figure 3:**
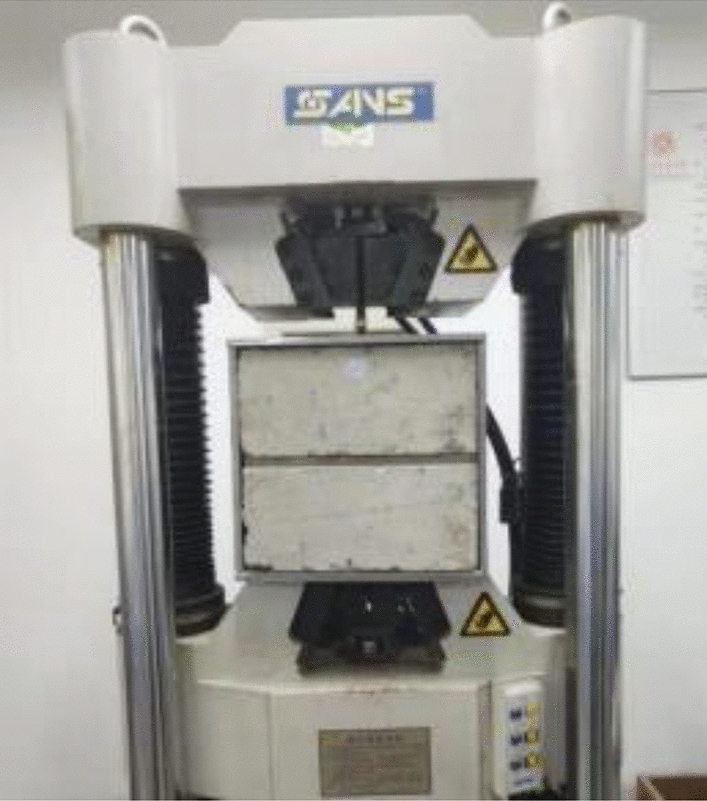
Test loading equipment.

A universal testing machine is used to conduct tensile strength tests on the samples set aside after each grouting cycle. The tests are performed under displacement control mode, with a loading rate of 1.0 mm/min, until the sample exhibits apparent damage or a sudden drop in the load-slip curve. In addition to recording the ultimate tensile strength, the load–displacement curve and the strain values of the steel bars are also documented. A representation of the testing loading apparatus is shown in Fig. 3.

## Results

### Experimental phenomena of microbial cement grouting process

The gaps between the concrete and the reinforcing steel bars are filled with calcium carbonate or a mixture of calcium carbonate and the medium, which are formed through the gradual mineralization of calcium carbonate. The filling process primarily undergoes four stages: flow, adhesion, bridging, and sealing. As the number of grouting cycles increases, the distribution of calcium carbonate on the steel bars gradually increases and becomes more uniform.

During the initial flow stage, the grout smoothly flows into the larger gaps between the concrete and the steel bars, and then flows out from the pores at the bottom of the steel bars. At this time, a brown liquid and white precipitate appear in the waste collector at the bottom. As the number of grouting cycles increases, the adhesion stage begins, where the formed calcium carbonate precipitate starts to adhere to the steel bars, the pore walls, and the added medium, although some of it gets washed to the bottom of the pores.This is followed by the bridging stage, where the original gaps are gradually divided into smaller gaps by the formed precipitate, slowing the flow of grout and accelerating the generation of precipitate. Finally, during the sealing stage, the small gaps are gradually sealed off as the number of calcium carbonate crystals increases, forming a sealing layer along the depth of the gaps and thus concluding the grouting process.

### Experimental phenomena of microbial cement anchoring steel bar pull-out test

**Figure 4:**
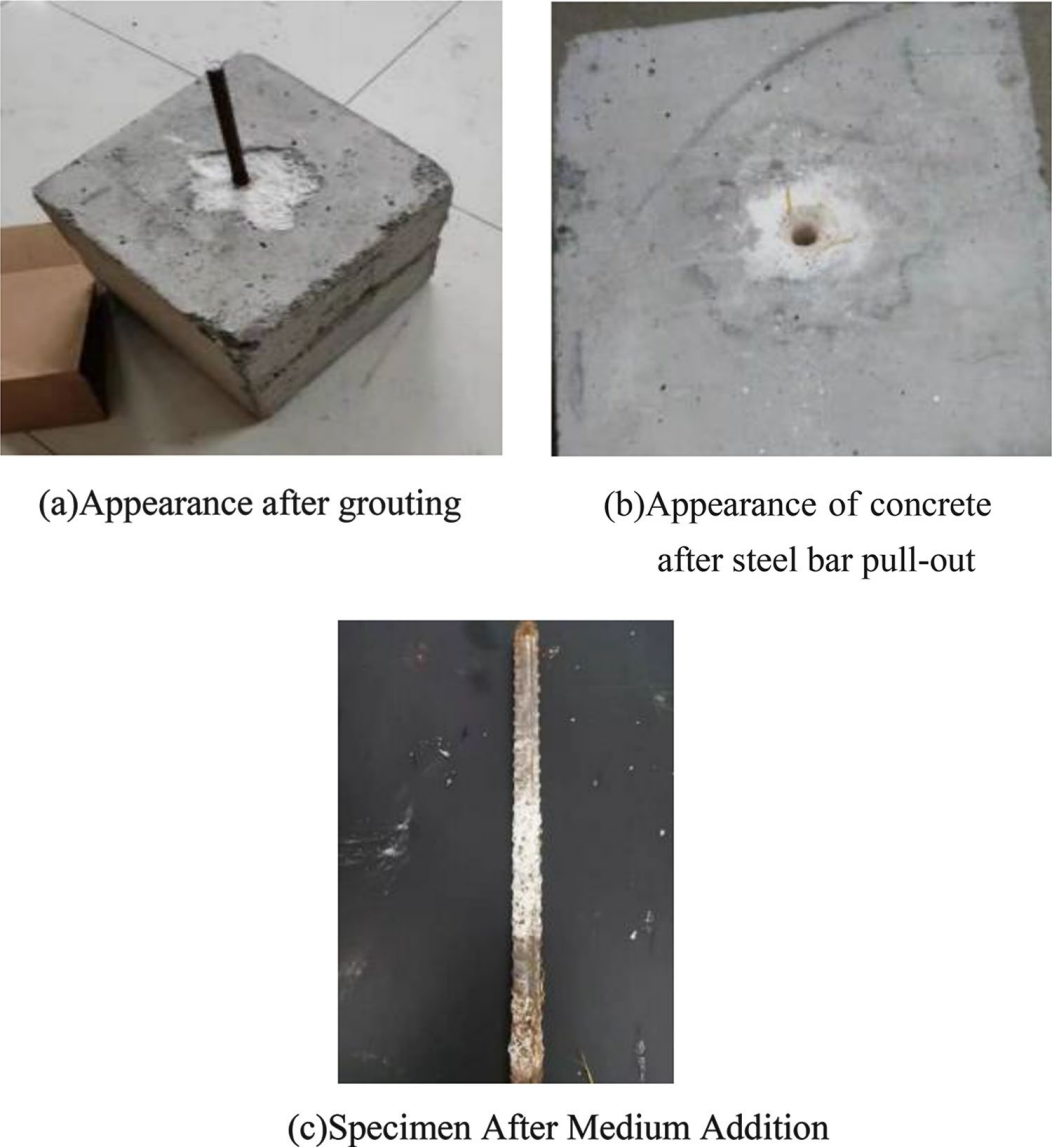
Pull-out test phenomenon of anchor steel bar.

After the four stages of microbial cement grouting, the anchoring of the steel bars is completed, as shown in Fig. 4a. A pull-out test is conducted on the anchored steel bars, which uniformly results in a pull-out failure at the interface between the steel bars and the concrete specimens, as depicted in Fig. 4b. Upon extraction, the distribution of calcium carbonate precipitate on the steel bars in the non-medium group is fairly uniform, with a higher buildup of calcium carbonate precipitate in the recesses of the steel bar ribs.

In contrast, the steel bars in the specimens with added medium experience friction from quartz sand during the pull-out process. This leads to the destruction of some of the calcium carbonate on the steel bars, creating a somewhat chaotic distribution, as shown in Fig. 4c. As the number of grouting cycles increases, the distribution of calcium carbonate on the steel bars gradually becomes more substantial. The mode of failure is consistently steel bar pull-out.

### Test results of resist pull-out bearing capacity of anchored steel bars

In order to identify the optimal grouting parameters, pull-out tests are conducted on anchored steel bars under varying experimental parameters. This yields the test results of the steel bar’s pull-out bearing capacity after each round of grouting anchoring.

#### Different calcium source concentrations

Pull-out tests were performed on three sets of test blocks with added calcium source concentrations of 0.5 mol/L, 1.0 mol/L, and 2.0 mol/L. The pull-out limit bearing capacities obtained from these tests are presented in Table 5.

**Table 5:** Resist pull-out ultimate bearing capacity of steel bars after each round of grouting with different calcium source concentrations (unit: N).

Number of grouting cycles	The calcium source concentration is 0.5 mol/L	The calcium source concentration is 1.0 mol/L	The calcium source concentration is 2.0 mol/L
3	230.32	289.14	311.23
4	389.11	434.03	576.02
5	530.37	687.76	575.83
6	703.23	687.23	
7	703.35		

Due to the small amount of calcium carbonate generated in the first 2 rounds of grouting, their anchoring ability to the steel bar was insufficient, and the value could not be recorded.

Table 5 reveals that as the number of grouting cycles increases, the anchoring pull-out bearing capacity of the reinforcement bars progressively heightens. However, after a certain number of grouting cycles, the increase in anchoring pull-out bearing capacity is no longer significant, and the borehole is nearly blocked. This suggests that by this point, calcium carbonate has essentially filled the voids in the borehole, achieving the densest state. The grouting cycle immediately preceding the maximum bearing capacity provides the most significant increase in the pull-out resistance of the reinforcement bars. This is due to the fact that the calcium carbonate precipitates generated from the early grouting cycles were insufficient to entirely block the borehole’s voids, causing the binder to not form as a whole, and hence a lower pull-out resistance. However, once the final grouting cycle’s precipitates block the voids, the circumferential wrapping force on the rebar increases, significantly boosting the anchoring effect.

The comparison group with the lowest concentration of calcium source in the grouting fluid shows the highest anchoring pull-out bearing capacity of the reinforcement bars. This happens because test pieces with high calcium source concentrations generate calcium carbonate precipitates more quickly and in larger quantities than those with lower concentrations, blocking the borehole with fewer grouting cycles. This leads to uneven distribution of calcium carbonate precipitates and correspondingly lower density. The contact area between the rebar and the borehole decreases, causing a reduction in anchoring capacity. Therefore, test pieces with higher calcium source concentrations require noticeably fewer grouting cycles, but result in lower anchoring pull-out bearing capacities of the reinforcement bars.

#### Filling medium in the pore space

When the calcium source concentration is 1 mol/L, and the particle size of the medium filled in the pores of the pores is 0–0.5 mm, 0.5–1 mm, and 1–2 mm, respectively, the pull-out ultimate bearing capacity of the anchored steel bars after each round of grouting is shown in the Table 6.

**Table 6:** Resist pull-out ultimate bearing capacity of anchored steel bars after each round of grouting with different medium particle sizes (unit: N).

Number of grouting cycles	The particle size is 0 ~ 0.5 mm	The particle size is 0.5 ~ 1 mm	The particle size is 1 ~ 2 mm
1	5762.35	6221.02	5534.53
2	9733.43	11,325.60	8447.23
3		18,892.32	12,870.51

Based on Table 6, we can conclude that for the comparison group with filled medium particle size, the anchoring pull-out bearing capacity of the steel bars reaches its maximum when the medium particle size is in the range of 0.5 ~ 1 mm. This is due to the approximately 2 mm gap between the borehole and the steel bar. When the medium particle size is in the range of 0 ~ 0.5 mm, the particle size is smaller than a quarter of the gap. This tends to form a rolling mechanism when there are more than three medium particles in the gap. For particle sizes in the 1 ~ 2 mm range, the medium tends to get stuck in the gap near the borehole opening during filling, making it difficult to fill the entire length of the borehole with the medium. However, when the medium particle size is in the range of 0.5 ~ 1 mm, it not only ensures the complete filling of the borehole but also reliably anchors the steel bar.

Comparing Tables 5 and 6, it’s evident that the ultimate pull-out bearing capacity of steel bars anchored in boreholes filled with quartz sand medium significantly increases compared to samples without a filling medium. Specifically, for samples with a medium particle size of 0.5 ~ 1 mm, the ultimate pull-out bearing capacity of the anchored steel bars can reach 18,892.32 N, which is 26 times higher than that of samples with the same calcium source concentration but without a filling medium. This is because the anchoring force on the steel bars in samples without a filling medium mainly comes from the chemical binding force provided by the precipitated calcium carbonate, which is not very effective in anchoring steel bars. However, for samples filled with quartz sand medium, the mixture of quartz sand and precipitated calcium carbonate forms a high-strength filler, which not only provides a binding effect but also offers significant friction and mechanical interlocking forces for steel bar anchoring, making it more effective.

Furthermore, adding medium to the sample boreholes significantly reduces the number of grouting cycles. When the medium particle size is in the range of 0 ~ 0.5 mm, only two grouting cycles are needed to achieve the best anchoring force on the steel bars. This is because after filling the medium, the borehole gap is divided into multiple small hole structures, providing a better environment for the deposition of calcium carbonate and facilitating the formation of “bridging” action".

#### Rebars with different shapes

The grouting solution with calcium source concentration of 1.0 mol/L was not filled with medium in the pores of the pores,and the ultimate bearing capacity of resist pull-out anchorage of steel bars with different shapes is shown in Table 7.

**Table 7:** Resist pull-out ultimate bearing capacity of anchored steel bars with different shapes (unit: N).

Cycle grouting times	3	4	5
Ribbed steel bar	289.14	434.03	687.76
Plain round steel bar	280.24	395.3	662.53

As can be seen from Table 7, for samples grouted with the same calcium source concentration, the pull-out bearing capacity of ribbed steel bars is greater than that of smooth round steel bars. This is due to the increased contact surface area and mechanical interlocking force provided by the ribbed steel bars in comparison to the smooth round steel bars, resulting in a higher pull-out bearing capacity.

### Load–displacement curve of anchor steel bar pull-out test

**Figure 5:**
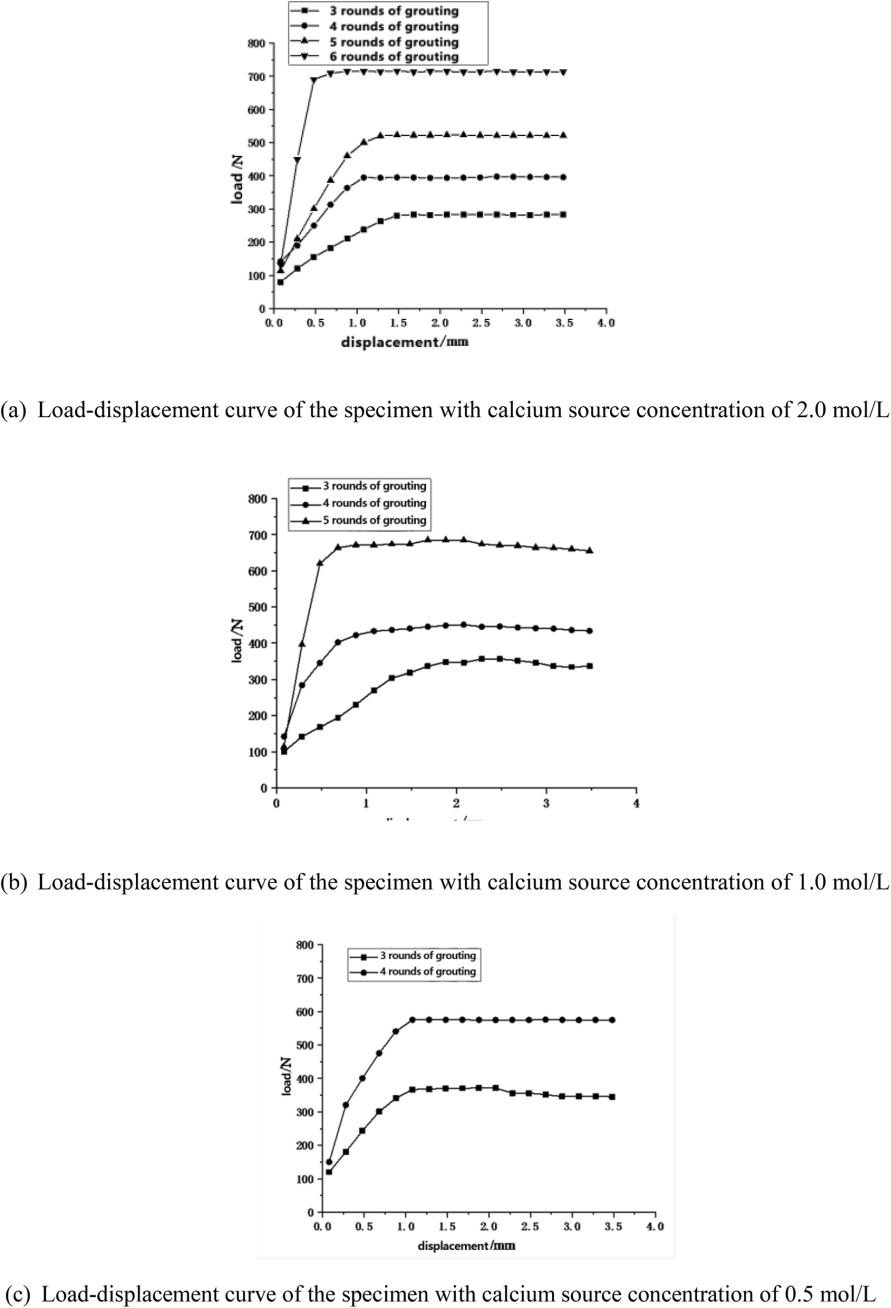
Load–displacement curve of the anchoring steel bar under the condition of unfilled medium in the tunnel void.

For the condition that the medium is not filled in the pore space, the load–displacement curve in the pull-out test of the anchored steel bar is shown in Fig. 5.

As illustrated in Fig. 5, the load–displacement curve of the anchored steel bars initially exhibits a linear elastic rising trend, where displacement increases proportionally with the load. Upon reaching the limit of its bearing capacity, the displacement continues to grow while the bearing capacity remains relatively stable. During this phase, the specimen is in a state of plastic deformation, with the steel bars beginning to slip. The effect of plastic deformation is significant, with the total amount of plastic deformation being approximately three to four times that of the elastic phase.

**Figure 6:**
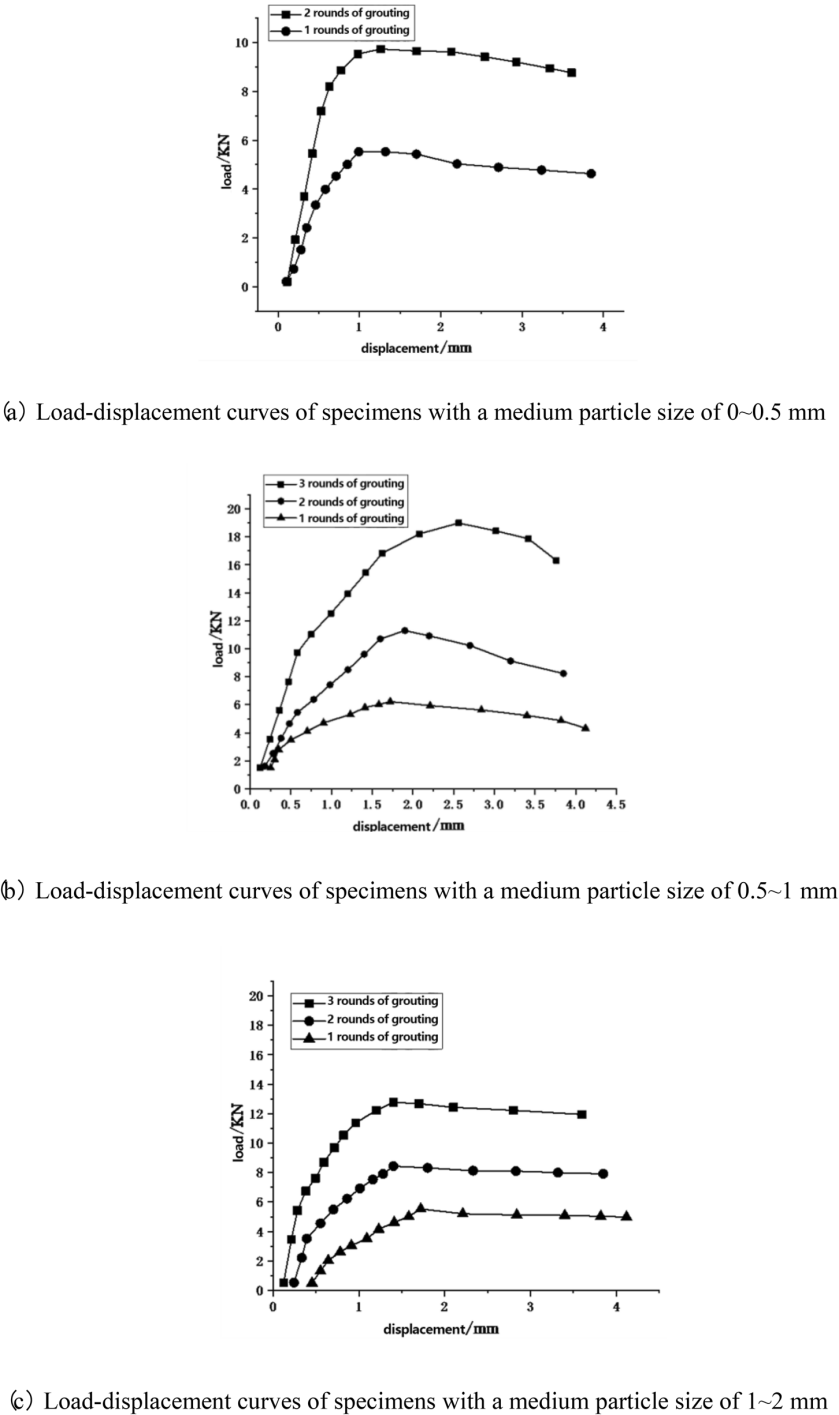
Load–displacement curve of anchored steel bar under the condition of filling medium in the pore space.

For the conditions where the borehole voids are filled with a medium, the load–displacement curve of the pull-out test for the anchored steel bars can be seen in Fig. 6.

As can be seen from Fig. 6, the load–displacement curve during the pull-out process of the anchored steel bars can be divided into three stages: the linear elastic rising stage, the limit bearing stage, and the stage of steel bar slippage and stress “softening”. This is because: the anchoring force of the steel bar is mainly provided by the chemical bonding force and friction of the mixture of calcium carbonate precipitation and quartz sand on the steel bar. In the early stage of bearing, damage has not occurred within the calcium carbonate and quartz sand, and the friction strength and bond strength have not been fully utilized, thus the rising slope remains constant. As the tensile load increases, it gradually reaches the limit bearing capacity, and the bond strength is first fully utilized. Microcracks start to intersect and penetrate each other, and potential sliding surfaces are gradually generated. At this point, the stress reaches the damage threshold, the friction strength begins to be fully utilized, and the bond strength starts to decline continuously, causing the curve slope to change and show distinct non-linear features. Entering the softening stage, the bearing capacity decreases. As the scale of crack penetration increases, a sliding surface forms, the friction strength decreases and tends to stabilize at the residual friction strength. At the same time, the bond strength continuously decreases until it is essentially lost.

### Load–strain curve of anchoring reinforcement

**Figure 7:**
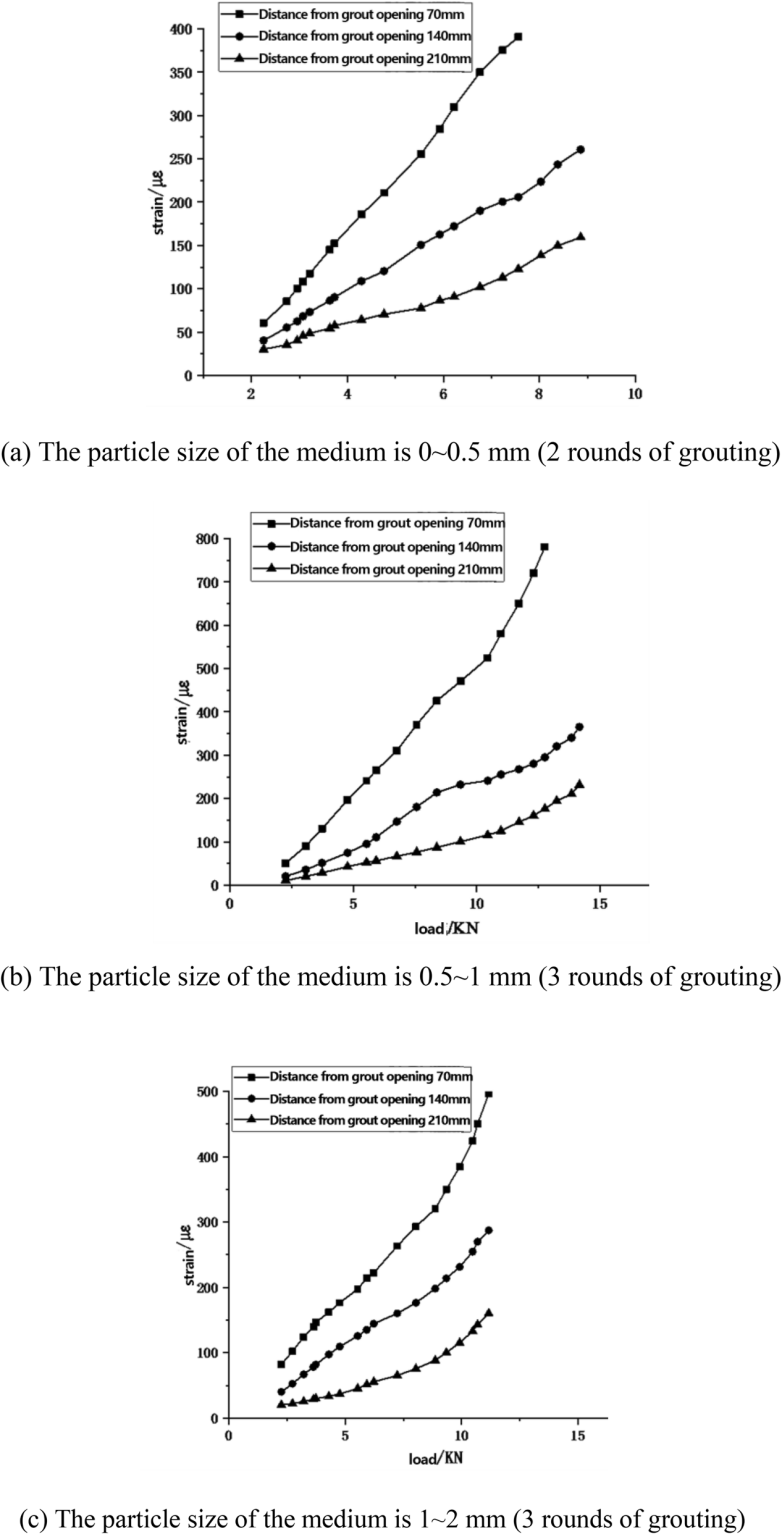
Load–strain curves at different positions of steel bars.

In order to obtain the distribution law of the stress of the anchored steel bar during the pull-out test of the anchored steel bar, the strain gauges were pasted at different positions of the anchored part of the steel bar under the test condition of the filling medium in the tunnel (70 mm, 140 mm and 210 mm near the grouting port, respectively). The load-strain test results of different anchoring positions of specimen reinforcement with a medium particle size of 0 ~ 0.5 mm (2 rounds of grouting), 0.5 ~ 1 mm (3 rounds of grouting) and 1 ~ 2 mm (3 rounds of grouting) were obtained by the strain gauge data collection box as shown in Fig. 7.

From Fig. 7, it is apparent that the stress in the steel bar increases with the rise in the pullout load. The stress in the steel bar near the grouting port is significantly higher than that in the sections farther away. This suggests that under the pullout force, the anchoring force of the steel bar near the grouting port is activated first. As the pullout force continues to grow, the stress in the steel bar located farther from the grouting port gradually rises and the anchoring force within this section is progressively stimulated. The anchoring force in the steel bar is transferred gradually from the tension end towards the far end.

## Analysis of the mechanism of microbial cement anchoring reinforcement bars

**Figure 8:**
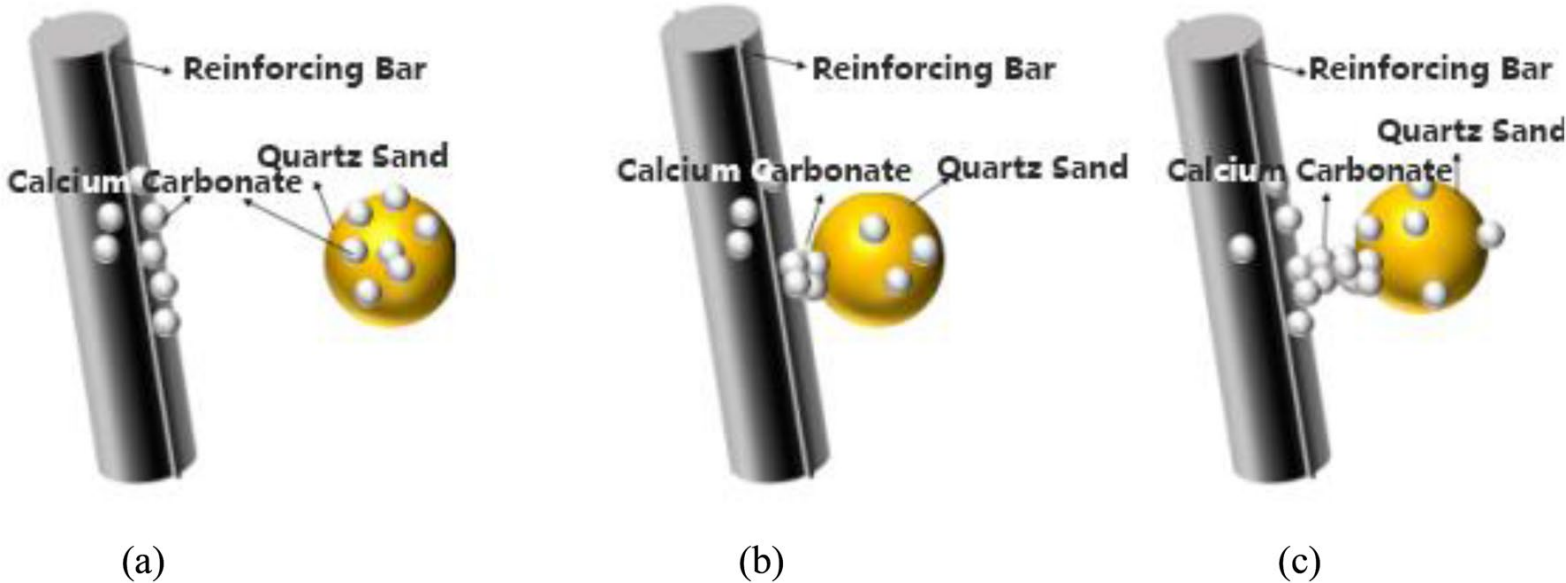
Schematic diagram of microbial cement grouting anchor reinforcement.

The process of anchoring reinforcement bars using microbial cement grouting can be divided into several stages, evolving with the increase in the number of grouting cycles. In the initial stage, due to the large pore size, negatively charged microbial cells get adsorbed onto the surface of quartz sand particles and the steel bars. When adequate nutrients (such as urea-CaCl_2_) are present in the grouting environment, microbial cement induces mineralization, leading to the formation of calcite, a binding substance, between the quartz sand particles and on their surface. This calcite adheres to the surface of the quartz sand particles and the steel bars, reducing the porosity and increasing the surface roughness, which in turn increases the internal friction angle between the quartz sand and the steel bar surfaces, as shown in Fig. 8a. As the number of grouting cycles increases, calcium carbonate crystals begin to adhere to the quartz sand near the steel bars, binding them into a whole with certain mechanical properties, as depicted in Fig. 8b. Finally, more calcium carbonate crystals form large clusters between quartz sand particles and the steel bars farther away, serving as a bridge and further enhancing the binding to the steel bars, as illustrated in Fig. 8c.

When the steel bars, anchored by microbial cement grouting, experience tension, they transfer the load to the calcium carbonate and quartz sand. These materials then further distribute the load to the concrete. The steel bar extraction process consists of three stages: adhesion, slippage, and softening. During the adhesion stage, the adhesive stress comes from both the chemical adhesion of the grout and the friction of the contact surface. Similarly to standard cement-based materials, the bond between calcium carbonate and quartz sand also provides constraint stress. As the force applied increases, the bonding strength between calcium carbonate crystals progressively deteriorates. However, some calcium carbonate remains on the quartz sand surface, while the failed calcium carbonate collaborates with quartz sand particles to provide friction for the steel bars. In the slippage stage, as the tensile load increases, the bonding strength gradually weakens, and the steel bars start to slip. At this point, the friction between the calcium carbonate and quartz sand on the steel bars becomes fully utilized, displaying apparent non-linear characteristics. Once the bonding is entirely lost, the calcium carbonate on the quartz sand surface increases its roughness, further reducing the adhesive strength and causing a softening phenomenon in the specimen. According to prior steel bar pull-out tests, the anchoring force of the grouted specimens cannot make the steel bars yield. The mode of failure is consistently steel bar pull-out.

## Conclusions

1. Regarding the comparison groups with different calcium source concentrations, the higher the calcium source concentration, the less the number of grouting cycles required for the specimen. However, this results in lower pull-out bearing capacity of the anchored steel bars.
2. For the comparison groups filled with media of different particle sizes in the borehole, the pull-out bearing capacity of the anchored steel bars in specimens filled with quartz sand is significantly higher than that in specimens without filling. Among them, the pull-out bearing capacity of the anchored steel bars in specimens with a medium particle size of 0.5 ~ 1 mm (1/4*δ* ~ 1/2*δ*) is the highest. In comparison groups with different steel bar shapes, ribbed steel bars have slightly higher pull-out bearing capacities than smooth round steel bars.
3. As the number of grouting cycles increases, the anchoring force of the steel bars continuously enhances. However, once a certain number of grouting cycles is reached, the pull-out bearing capacity of the steel bars in each group almost stops increasing. The number of grouting cycles obtained at this time can be regarded as the optimal number of grouting cycles. The grouting cycle before reaching the optimal number contributes the most to the increase in the anchoring force of the steel bars. Filling the boreholes of the specimens with media significantly reduces the optimal number of grouting cycles.
4. Specimens without filling in the borehole exhibit better plasticity after reaching the ultimate bearing capacity, with the total plastic deformation amount being approximately 3 ~ 4 times that of the elastic stage. Specimens filled with media show a significant softening phenomenon after reaching the ultimate bearing capacity.
5. During the pull-out process of the anchored steel bars, the strain growth of the steel bars at the measuring points close to the grouting port is relatively fast, and the anchoring force of the steel bars gradually transfers from the tensile end to the far end.
6. When the rebar anchored by microbial cement grout is subjected to tension, the anchored rebar transfers the load it carries to the calcium carbonate and quartz sand, which in turn pass the load onto the concrete. The process of rebar extraction can be divided into three phases: the bonding stage, slipping stage, and softening stage.

## Data Availability

All data generated or analysed during this study are included in this published article [and its supplementary information files].

## References

[1] Kuang Z, Zheng G, Jiao X (2019). Experiment on the effect of insufficient grouting on the mechanical properties of steel bar-sleeve connection. J. Tongji Univ. (Nat. Sci. Edn.).

[2] Ferris, F. G., & Setehmeir, L. G. Bacteriogenic mineral plugging. 1992. United States patent 664769.

[3] Gollapudi UK, Knutson CL, Bang SS (1995). A new method for controlling leaching through permeable channels. Chemosphere.

[4] Soon NW, Lee LM, Khun TC (2013). Improvements in engineering properties of soils through microbialinduced calcite precipitation. KSCE J. Civ. Eng..

[5] Lee LM, Ng WS, Tanaka Y (2013). Stress-deformation and compressibility responses of bio-mediated residual soils. Ecol. Eng..

[6] Soon NW, Lee LM, Khun TC (2014). Factors affecting improvement in engineering properties of residual soil through microbial-induced calcite precipitation. J. Geotech. Geoenviron. Eng..

[7] Kim D, Park K (2013). Effects of ground conditions on microbial cementation in soils. Materials.

[8] Zhao Q, Li L, Li C (2014). Factors affecting improvement of engineering properties of MICP-treated soil catalyzed by bacteria and urease. J. Mater. Civ. Eng..

[9] Hill DD, Sleep BE (2022). Effects of biofilm growth on flow and transport through a glass parallel plate fracture. Contam. Hydrol..

[10] Metayer-Levrel GL, Castanier S, Orial G (1999). Applications of bacterial carbonatogenesis to the protection and regeneration of limestones in buildings and historic patrimony. Sediment Geol..

[11] Wiktor V, Jonkers HM (2011). Quantification of crack-healing in novel bacteria-based self-healing concrete. Cem. Concr. Compos..

[12] Whiffin VS, Van Paassen LA, Harkes MP (2007). Microbial carbonate precipitation as a soil improvement technique. Geomicrobiol. J..

[13] Zhang X, Qian C (2021). Effects of the crack geometric features on the probability density of spherical healing agent particles in concrete. Constr. Build. Mater..

[14] Qian CX, Pan QF, Wang RX (2010). Cementation of sand grains based on carbonate precipitation induced by microorganism. Sci. China Technol. Sci..

[15] Jia, Q., & Zhang, X. Microbial grouting method and structure for connecting longitudinal reinforcement of concrete members: CN,201910063263.X. 2019.5.21.

[16] Jia Q, Chen X, Sun Z, Xing J (2015). Experimental study on microbial-induced deposition of calcium carbonate to increase yield. J. Shandong Jianzhu Univ..

